# The combination of autofluorescence endoscopy and molecular biomarkers is a novel diagnostic tool for dysplasia in Barrett's oesophagus

**DOI:** 10.1136/gutjnl-2013-305975

**Published:** 2014-04-10

**Authors:** Massimiliano di Pietro, David F Boerwinkel, Mohammed Kareem Shariff, Xinxue Liu, Emmanouil Telakis, Pierre Lao-Sirieix, Elaine Walker, George Couch, Leanne Mills, Tara Nuckcheddy-Grant, Susan Slininger, Maria O'Donovan, Mike Visser, Sybren L Meijer, Philip V Kaye, Lorenz Wernisch, Krish Ragunath, Jacques J G H M Bergman, Rebecca C Fitzgerald

**Affiliations:** 1 Medical Research Council, Cancer Unit, University of Cambridge, Cambridge, UK; 2 Department of Gastroenterology, Academic Medical Centre, Amsterdam, The Netherlands; 3 Digestive Disease Centre, NIHR Biomedical Research Unit, Nottingham University Hospitals NHS trust, Nottingham, UK; 4 Department of Pathology, Academic Medical Center, Amsterdam, The Netherlands; 5 Department of Pathology, Nottingham University Hospitals NHS Trust, Nottingham, UK; 6 Biostatistics Unit, Medical Research Council, Cambridge, UK

**Keywords:** Barrett's Oesophagus, Oesophageal Cancer, Surveillance, Endoscopy, Dysplasia

## Abstract

**Objective:**

Endoscopic surveillance for Barrett's oesophagus (BO) is limited by sampling error and the subjectivity of diagnosing dysplasia. We aimed to compare a biomarker panel on minimal biopsies directed by autofluorescence imaging (AFI) with the standard surveillance protocol to derive an objective tool for dysplasia assessment.

**Design:**

We performed a cross-sectional prospective study in three tertiary referral centres. Patients with BO underwent high-resolution endoscopy followed by AFI-targeted biopsies. 157 patients completed the biopsy protocol. Aneuploidy/tetraploidy; 9p and 17p loss of heterozygosity; *RUNX3*, *HPP1* and *p16* methylation; p53 and cyclin A immunohistochemistry were assessed. Bootstrap resampling was used to select the best diagnostic biomarker panel for high-grade dysplasia (HGD) and early cancer (EC). This panel was validated in an independent cohort of 46 patients.

**Results:**

Aneuploidy, p53 immunohistochemistry and cyclin A had the strongest association with dysplasia in the per-biopsy analysis and, as a panel, had an area under the receiver operating characteristic curve of 0.97 (95% CI 0.95 to 0.99) for diagnosing HGD/EC. The diagnostic accuracy for HGD/EC of the three-biomarker panel from AFI+ areas was superior to AFI− areas (p<0.001). Compared with the standard protocol, this panel had equal sensitivity for HGD/EC, with a 4.5-fold reduction in the number of biopsies. In an independent cohort of patients, the panel had a sensitivity and specificity for HGD/EC of 100% and 85%, respectively.

**Conclusions:**

A three-biomarker panel on a small number of AFI-targeted biopsies provides an accurate and objective diagnosis of dysplasia in BO. The clinical implications have to be studied further.

**Significance of this study**

**What is already known on this subject?**

- Endoscopic surveillance for Barrett's oesophagus with random 4-quadrant biopsies has limitations including sampling error and subjectivity of dysplasia diagnosis.
- Endoscopic red-flag techniques, such as autofluorescence imaging (AFI), can improve diagnostic yield, but cannot not replace random biopsies.
- Some molecular biomarkers correlate with the dysplasia status of patients with Barrett's oesophagus.

**What are the new findings?**

- We have validated 8/9 molecular biomarkers in an independent laboratory for the first time. Of these a small 3-biomaker panel including p53 immunohistochemistry, cyclin A and aneuploidy had the best correlation with prevalent dysplasia.
- AFI positivity correlated with molecular biomarker status even in the absence of dysplasia.
- To determine the overall patient dysplasia status the 3-biomaker panel applied to AFI-targeted biopsies had equivalent sensitivity for high grade dysplasia and early cancer compared to the current clinical Seattle protocol, with a 4.5 fold reduction in the number of biopsies required.

**How might it impact on clinical practice in the foreseeable future?**

- The combination of molecular biomarkers and AFI may a useful tool for surveying patients with long segment of Barrett's oesophagus as it reduces the number of biopsies needed without loss of diagnostic accuracy.

## Introduction

Barrett's oesophagus (BO) is the only recognised precursor to oesophageal adenocarcinoma (OAC)^1^ whose incidence has dramatically increased over the last 40 years in the Western world.^2–4^ Despite the contradictory evidence on the benefit of endoscopic monitoring for BO,^5^ ^6^ all specialist societies recommend surveillance with a protocol entailing four-quadrant random biopsies every 2 cm (Seattle protocol).^7^ ^8^ However, this protocol is time consuming and invasive with subsequent poor adherence by endoscopists.^9^ ^10^ In addition, dysplasia and early cancer (EC) can be inconspicuous, leading to sampling error.^11^ Hence, clinical justification and cost-effectiveness of endoscopic surveillance has been questioned, especially following recent evidence that the cancer risk in BO is lower than previously thought.^12^ ^13^

Autofluorescence imaging (AFI) can improve recognition of inconspicuous dysplasia.^14–16^ Two previous cross-over studies have shown that, although AFI improves detection of dysplasia, it has a high false-positive rate (>60%) and its accuracy was not sufficient to replace the Seattle protocol.^14^ ^15^

An important limitation of endoscopic surveillance is the significant interobserver variability in the histopathological diagnosis of dysplasia.^17^ ^18^ This could be improved by molecular biomarkers that could provide more objective scores and cut-off values. Some of these, such as overexpression of p53 and cyclin A, methylation of specific genes, loss of heterozygosity (LOH) at the 17p and 9p loci and DNA ploidy abnormalities, associate with dysplasia.^19–24^ However, with the exception of p53 expression, these biomarkers have only been tested by single groups in single, retrospective cohorts of patients and this has hampered their translation into clinical practice. In addition, there is a lack of prospective studies testing multiple biomarkers in the same prospective cohort to identify the smallest, clinically applicable panel with the highest diagnostic accuracy. In this study, we used AFI to enrich our biopsy samples prior to biomarker analysis since we hypothesised that these areas may correlate with a field of molecular abnormalities indicative of the overall dysplasia status of the patient and hence reduce sampling bias.

Therefore, the primary aim of this prospective study was to compare the accuracy of a panel of molecular biomarkers on AFI-directed biopsies with the conventional quadrantic biopsies every 2 cm for the diagnosis of high-grade dysplasia (HGD) and EC. The secondary aims were (i) assessment of diagnostic accuracy for the biomarkers for any grade of dysplasia and (ii) validation of a large panel of biomarkers in an independent prospective study by an independent laboratory.

## Methods

### Patients and setting

The study was approved by the Cambridgeshire 2 Research Ethics Committee (09/H0308/118). For the generation of the biomarker panel, a training cohort of 175 patients was recruited prospectively between April 2009 and October 2011 from three centres. For the validation of the panel, an independent cohort of 46 patients was prospectively recruited between March 2012 and April 2013 at a single institution (Cambridge). Inclusion criteria were age >18 years, known BO with minimum length of C≥2 or C<2M≥4 according to the Prague classification,^25^ referral for evaluation of dysplastic BO or follow-up postendoscopic resection for HGD/EC. Short segments of <2 cm were excluded due to the excess of AFI-false positivity at the gastro-oesophageal junction and since sampling error is not such an issue for these cases.^26^ Exclusion criteria were oesophagitis (Los Angeles grade ≥B); previous upper gastrointestinal (GI) surgery (with the exception of Nissen fundoplication) or known upper-GI tract abnormality (eg, pharyngeal pouch); coagulopathy or anticoagulant/antiplatelet therapy for high-risk conditions; active or severe cardiopulmonary disease or liver disease; dysphagia; and special communication needs.

### Endoscopic procedure

Patients were endoscoped with FQ260Z endoscopes (Olympus Inc, Tokyo, Japan) as previously described.^14^ Five endoscopists performed the procedures, but in order to uniform the interpretation of the AFI signal, the three endoscopists with less experience in AFI performed at least 30 procedures prior to the study under supervision of more experienced endoscopists (JJGHMB and KR). The oesophagus was inspected first by white light high-resolution endoscopy (HRE) to detect visible lesions (see online supplementary figure S1A). Then, in AFI mode, AFI-positive (AFI+) areas (violet-purple in colour) were carefully mapped. A representative AFI-negative (AFI–) area (green colour) was then selected as a negative control (see online supplementary figure S1B). In patients who had diffuse patchy AFI positivity throughout the BO (n=8), an AFI– area was not selected, but they were included in the per-biopsy (figure 2) and final per-patient analysis (figure 4).

### Biopsy and histology

Each AFI+ area and one AFI− area were biopsied for biomarker analysis and histopathology (see online supplementary figure S1A). Up to a maximum of four AFI+ areas were included in the research protocol; however, small AFI+ areas (<1 cm) within 1 cm from the gastro-oesophageal junction were excluded due to the well-known false positivity in close proximity to the gastric folds.^27^ Where possible (depending on the size of the AFI+ area), a maximum of three biopsies were taken in the following order of priority: one biopsy in formalin, one snap frozen biopsy in 10% dimethysulfoxide (DMSO) and one biopsy snap frozen dry. For AFI+ areas larger than 1 cm, two or more biopsies were taken for histology to minimise sampling error, whereas AFI+ areas with a complex shape were considered as multiple AFI+ areas (see online supplementary figure S1B). Histopathological assessment of each AFI-targeted area relied on biopsies stored in formalin. Random biopsies were then taken according to the Seattle protocol. The histology was assessed by an expert GI pathologist at the respective participating centre according to the Vienna classification.^28^ All dysplastic cases, including indefinite for dysplasia (ID), were then reviewed by a second pathologist, with random pairing of the initial pathologist with one of the pathologists from the other centres. Four pathologists took part in initial assessment, but only three participated in the reviewing process. In case of disagreement, consensus was reached through a review process, and this diagnosis was considered as the final histological outcome. Cases with ID without definite dysplasia after consensus review were regarded as non-dysplastic (NDBO). In accordance with the Vienna classification cases of HGD, carcinoma in situ and intramucosal adenocarcinoma were grouped together (HGD/EC) as they represent a common endpoint for endoscopic therapeutic intervention.^7^ ^29^ For the purpose of the biomarker analysis, all biopsies taken using the AFI mode were regarded as AFI-targeted (AFI+ and AFI−). With regards to the overall per-patient histopathological diagnosis, we considered diagnoses made using (a) the current clinical standard (Seattle protocol, ie, biopsies on areas visible on HRE+ quadrantic random biopsies) and (b) the overall histology (a+ histology on AFI-targeted biopsies).

### Biomarker analyses

All molecular analyses were done at the Medical Research Council, Cancer Unit (Cambridge, UK). The panel of molecular biomarkers analysed in the training cohort (nine-biomarker panel) comprised aneuploidy, G2/tetraploidy, LOH at 9p and 17p loci, hypermethylation of *p16*, *RUNX3*, *HPP1* and immunohistochemistry (IHC) for p53 and cyclin A. Details of the molecular analyses are provided in the online supplementary material.

### Sample size and power

Previous data suggest that single biomarkers have a sensitivity for HGD/EC varying between 60 and 87%; however, we assumed that, when combined into a panel, biomarkers could have 90–95% sensitivity and 85–95% specificity. For a sensitivity of 90%, we calculated that to have an accuracy of 10% (95% CI ±10%) we needed to recruit at least 35 patients with HGD/EC.

### Statistics

For the purpose of the per-biopsy analysis, each AFI-targeted area was classified with (1) AFI status (AFI+ or AFI−), (2) binary outcome of the nine biomarkers and (3) histological diagnosis. c^2^ tests were used to compare differences between groups. A p value <0.05 was considered statistically significant. Interobserver agreement was assessed by κ statistics. For the pathological diagnosis, agreement among three expert pathologists for a diagnosis of any grade of dysplasia was expressed as weighted κ value. For the endoscopic location of AFI-targeted areas, the agreement was expressed as κ value; agreement among endoscopists was defined as their specification of the epicentre of the AFI+ area within 30° of the endoscopic view. For this analysis, 20 random AFI pictures were selected by two authors (DFB and MKS) and assessed by three authors (MDP, KR and JJGHMB).

The statistical analysis consisted of three stages: (1) per-biopsy analysis (correlation between biomarkers and histological outcome in individual targeted areas); (2) per-patient analysis (correlation between overall biomarker result and overall histological outcome in individual patients); and (3) comparison between AFI+ versus AFI− areas (comparative analysis of biomarker diagnostic accuracy for dysplasia in biopsies from AFI+ vs AFI− areas).

To obtain means, medians and SDs for various statistics, we relied on two types of simulations, multiple imputation (MI) and bootstrap resampling. Missing values were imputed by MI as it is generally preferred to impute values than to drop samples with missing values.^30^ ^31^ The only assumption for the MI procedure is that the missingness pattern does not depend on missing values, but it may depend on observed values.^32^ Since missing values are mostly related to the size of AFI+ areas, we assumed that this correlation could be captured in the observed biomarker values and missing values imputed. The imputation model included the nine biomarkers and the histological diagnosis as variables. Five independent data sets were imputed, and they were included in the analysis together with the original database, where missing values were removed. The variation in the imputed values between the MI data sets reflects uncertainty about the missing values.

Bootstrap resampling was used to obtain estimates of parameters and their SEs from finite sample sets to allow conclusions to be drawn about the population in general.^33^ B-artificial sample data sets were obtained from the original set of samples, where B is a suitably large number (in the hundreds or thousands). Each artificial data set was drawn randomly with equal size to the original data set. After selection into the bootstrap set, samples were put back into the original pool and could be selected again. Multiple rounds of a random selection of subsamples were performed and the calculated accuracy of the parameter of interest was validated in the remaining subsample. This prevented inflating the diagnostic performance of biomarkers by applying the model to the same data set from which it was developed (overfitting). Further details of these analyses are provided in the online supplementary material.

Details of the statistical methodology used for the generation and the validation of the biomarker panel are described in the online supplementary material.

All the analyses were performed using SPSS V.19.0 (SPSS Inc, Chicago, Illinois, USA) and R V.2.15.2.

## Results

Of the 175 participants recruited as part of the training cohort, 18 were excluded because of either a lack of biopsies for laboratory tests (n=4) or breach of biopsy protocol (eg, biopsy mislabelling, no research biopsy in AFI+ areas taken, no random biopsies taken) (n=14). From the remaining 157 participants, we obtained 373 AFI-targeted biopsies (AFI+:AFI−=230:143), which were processed for biomarkers and included in the per-biopsy analysis. The validation cohort consisted of 46 patients, from which 155 AFI-targeted biopsies were taken (AFI+:AFI−=108:47). There were no significant differences between the two cohorts in the demographics and histological outcomes as well as characteristics of AFI+ areas; however, the validation cohort had a slightly higher number of AFI+ areas per patient (table 1). The overall interobserver agreement among three expert pathologists for a diagnosis of any grade of dysplasia was ‘moderate’ (weighted κ value 0.56, SE 0.055). With reference to the interpretation of the AFI signal for targeting biopsies, the overall agreement for locating the area of interest among observers was ‘good’ (κ value 0.68, SE 0.104) and the interobserver variability did not change significantly when the agreement between two expert endoscopists was compared with the agreement between expert and non-expert endoscopists.

**Table 1:** Demographics, histological stage and endoscopic characteristics of AFI+ areas for patients included in training and validation cohorts

	Training cohort	Validation cohort	p Value
Variables			
Number of patients	157	46	N/A
Male: female (%)	79:21	93:7	0.06
Mean age (range)	66.4 (35–87)	68.7 (35–84)	0.23
Mean length of BO in cm (range)	7.3 (2–17)	7.6 (3–18)	0.85
Histological diagnosis			
NDBO	99 (63%)	22 (56.4%)	0.24
LGD	21 (13.4%)	8 (20.5%)	
HGD	24 (15.3%)	3 (7.7%)	
EC	13 (8.3%)	6 (15.4%)	
Endoscopic features			
Number of AFI+ areas	229	108	N/A
AFI+ areas visible on HRE	28.4%	21%	0.15
AFI+ areas with HGD/EC	21.1%	15.4%	0.32

AFI, autofluorescence imaging; EC, early cancer; HGD, high-grade dysplasia; HRE, high-resolution endoscopy; LGD, low-grade dysplasia; NDBO, non-dysplastic Barrett's oesophagus.

### Association of biomarkers with dysplasia (per-biopsy analysis)

**Figure 1:**
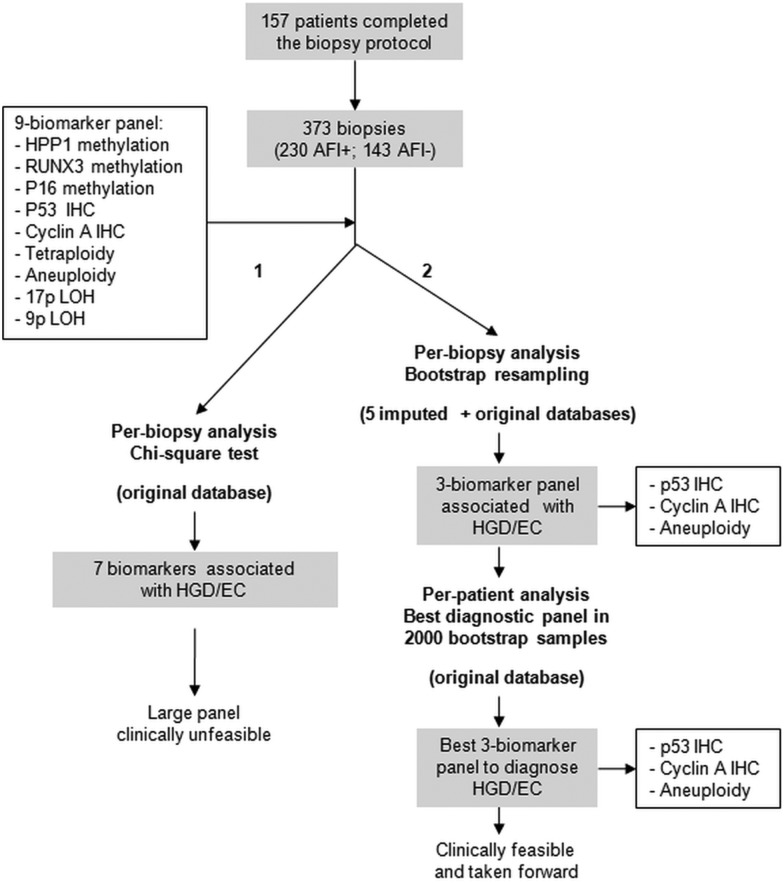
** **Strategy for the generation of the biomarker panel. χ^2^ test showed that from the initial panel of nine biomarkers seven significantly associated with a diagnosis of high-grade dysplasia/early cancer (HGD/EC) in the corresponding biopsy (arm 1—left side). To identify a small biomarker panel, bootstrap resampling on five imputed and one original database was applied (arm 2—right side). Both per-biopsy and per-patient analyses identified the same three-biomarker panel as the best diagnostic panel for HGD/EC.

We first sought to identify a small biomarker panel with the best diagnostic accuracy for prevalent dysplasia. To this end, we identified nine biomarkers that had the most robust correlation with dysplasia in BO based on published phase III and IV clinical studies.^19–24^ Figure 1 shows the strategy used for the generation of the panel. The nine biomarkers were tested in the AFI-targeted biopsies from the training cohort to assess their association with dysplasia on a per-biopsy basis. As shown in table 2, all of the biomarkers associated with the presence of confirmed dysplasia, with the exception of 9p LOH (p16). When restricting the analysis to the association between biomarkers and HGD/EC, we found similar results, in that only tetraploidy and 9p LOH lacked statistical significance.

**Table 2:** Association of biomarkers with dysplasia in the per-biopsy analysis

Biomarker	Missing values (%)	Biomarker outcome	HGD/EC			Any dysplasia		
			No	Yes	p Value	No	Yes	p Value
HPP1 methylation	19.1	Negative	59 (22.9%)	3 (7.1%)	0.02	56 (24.0%)	6 (9.0%)	<0.01
		Positive	199 (77.1%)	39 (92.9%)		177 (76.0%)	61 (91.0%)	
RUNX3 methylation	19.1	Negative	102 (39.5%)	6 (14.3%)	<0.01	97 (41.6%)	11 (16.4%)	<0.01
		Positive	156 (60.5%)	36 (85.7%)		136 (58.4%)	56 (83.6%)	
p16 methylation	19.1	Negative	143 (55.4%)	12 (28.6%)	<0.01	132 (56.7%)	23 (34.3%)	<0.01
		Positive	115 (44.6%)	30 (71.4%)		101 (43.3%)	44 (65.7%)	
p53 IHC	13.2	Negative	196 (70.5%)	3 (6.8%)	<0.01	189 (76.8%)	10 (13.2%)	<0.01
		Positive	82 (29.5%)	41 (93.2%)		57 (23.2%)	66 (86.8%)	
Cyclin A IHC	14.0	Negative	238 (85.0%)	8 (20.5%)	<0.01	223 (88.8%)	23 (33.8%)	<0.01
		Positive	42 (15.0%)	31 (79.5%)		28 (11.2%)	45 (66.2%)	
Tetraploidy	19.7	Negative	182 (69.2%)	20 (57.1%)	0.15	171 (72.2%)	31 (50.8%)	<0.01
		Positive	81 (30.8%)	15 (42.9%)		66 (27.8%)	30 (48.2%)	
Aneuploidy	19.7	Negative	232 (88.2%)	10 (28.6%)	<0.01	216 (91.1%)	26 (42.6%)	<0.01
		Positive	31 (11.8%)	25 (71.4%)		21 (8.9%)	35 (57.4%)	
17p LOH	27.4	Negative	112 (49.3%)	7 (16.7%)	<0.01	107 (51.9%)	12 (19.0%)	<0.01
		Positive	115 (50.7%)	35 (83.3%)		99 (48.1%)	51 (81.0%)	
9p LOH	28.5	Negative	43 (19.2%)	5 (12.2%)	0.29	40 (19.5%)	8 (13.3%)	0.27
		Positive	181 (80.8%)	36 (87.8%)		165 (80.5%)	52 (86.7%)	

Each p value is obtained from a χ^2^ test on the 2×2 table to its left. Any dysplasia refers to combination of low-grade dysplasia (LGD), HGD and EC.

EC, early cancer; HGD, high-grade dysplasia; IHC, immunohistochemistry; LOH, loss of heterozygosity.

**Figure 2:**
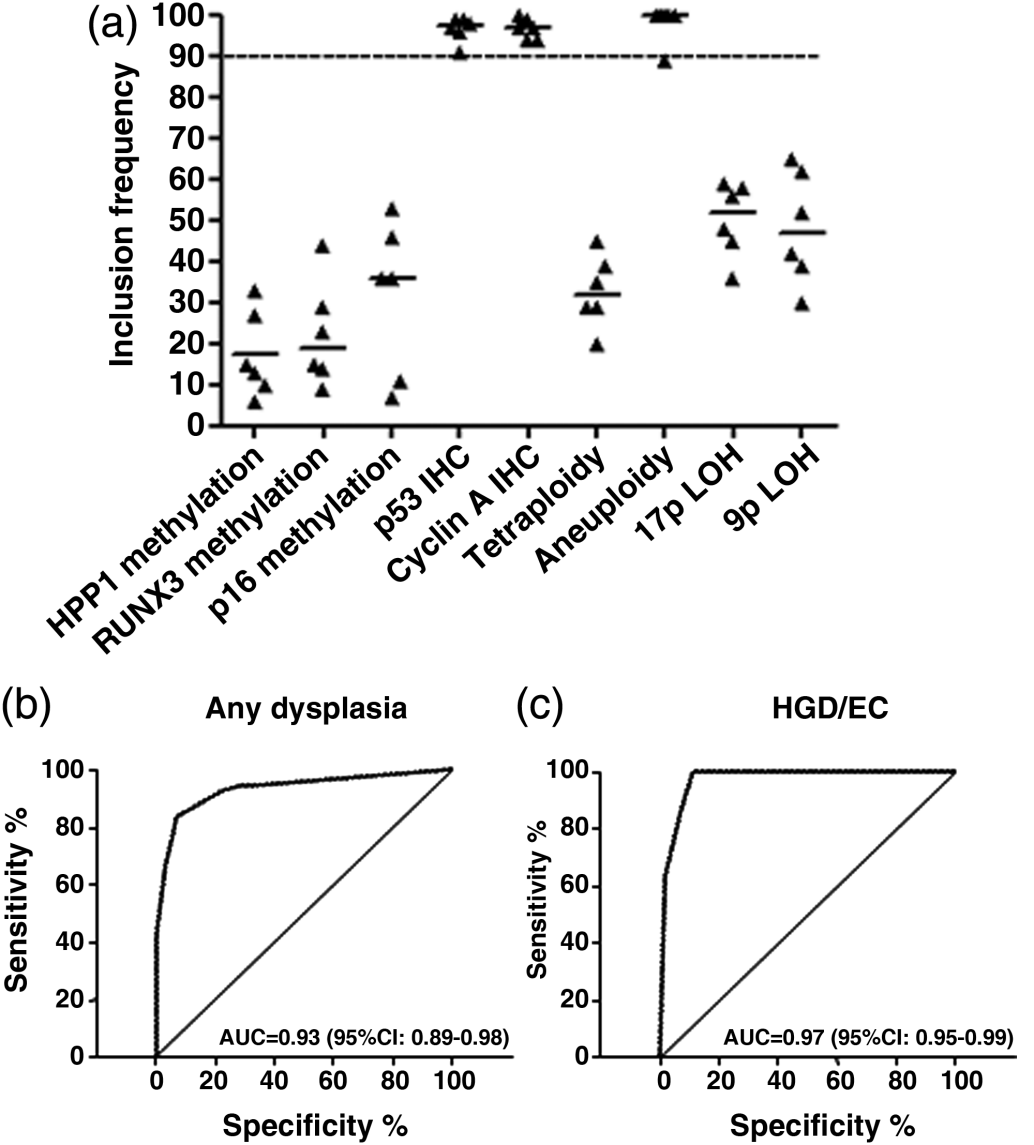
A three-biomarker panel including p53 immunohistochemistry (IHC), cyclin A IHC and aneuploidy has high diagnostic accuracy for dysplasia. (A) Inclusion frequencies of the nine biomarkers in 100 bootstrap samples for each MI database (n=5) and the original database (n=1). A stringent cut-off of 90 for the median over all six databases was used to select the best biomarkers. p53 IHC, cyclin A IHC and aneuploidy had median inclusion frequency above the threshold. (B,C) Area under the curve (AUC) for the diagnosis of any grade of dysplasia (B) and high-grade dysplasia/early cancer (HGD/EC) only (C) was calculated using the panel of biomarker selected in (A).

Since large numbers of biomarkers are difficult to apply in the clinical setting, we used a strict statistical methodology to identify a small biomarker panel. The data set had 20% of missing values as some AFI+ areas were not sufficiently large to allow three biopsies as per protocol (table 2). We used bootstrap resampling on the original database as well as on five imputed databases to account for missing values. This statistical methodology has been previously developed to handle data sets with up to 50% of missing data in clinical studies.^34^ We concentrated on the highest histological outcome for this analysis (HGD/EC) as this is what currently triggers therapeutic decisions according to clinical guidelines. We found that two IHC markers (p53 and cyclin A) and aneuploidy had the highest rate of inclusion in best bootstrap models (figure 2A), and for this reason they were selected to form a three-biomarker panel. On a per-biopsy analysis, this panel had area under the curves (AUCs) of 0.93 (95% CI 0.89 to 0.98) and 0.97 (95% CI 0.95 to 0.99) for a diagnosis of any grade of dysplasia and HGD/EC, respectively (figure 2B,C). These data showed that these three biomarkers have the strongest association with dysplasia and could be combined into a panel to aid clinical diagnosis.

### Association between molecular biomarkers and AFI status

**Figure 3:**
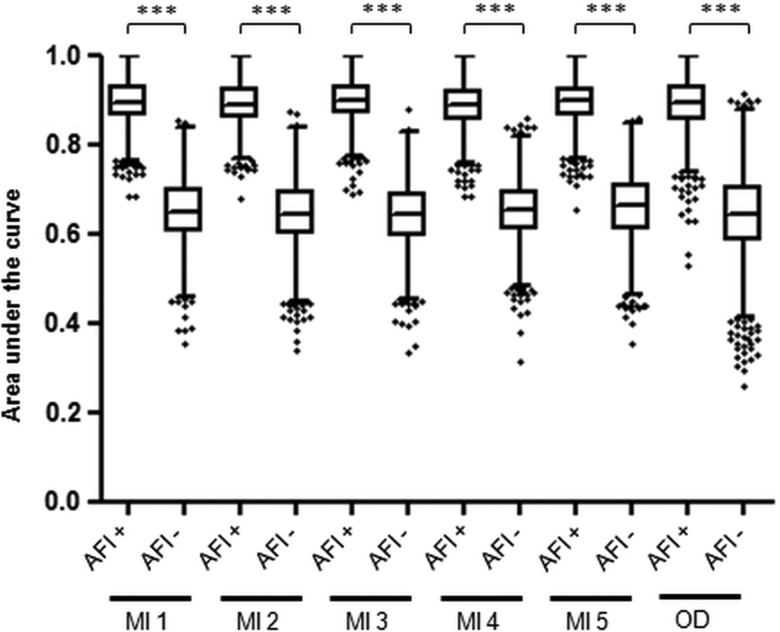
Diagnostic accuracy for high-grade dysplasia/early cancer (HGD/EC) of the three-biomarker panel assessed on autofluorescence imaging-positive (AFI+) areas and AFI− areas. This analysis was performed on the 114 patients with biopsies available on both AFI+ and AFI− areas, after exclusion of patients without AFI positivity (n=35) and those with diffuse AFI positivity (n=8). The area under the curve (AUC) for a diagnosis of overall HGD/EC was calculated using the three-biomarker panel from AFI+ areas and AFI− areas in 2000 bootstrap samples from five imputed databases (MI) and the original database (OD). In this plot, each box represents the median AUC with first and third quartiles for the bootstrap samples of each group and the whiskers include data within 1.5 IQR of the upper and lower quartile. Outliers are depicted separately. *** indicates p value <0.001.

In order to translate the diagnostic accuracy for dysplasia of the biomarker panel from a single biopsy to a per-patient level, we determined that the biopsies should be targeted using an imaging tool. Hence, we then asked whether AFI positivity correlates with molecular abnormalities. We first analysed the association between individual biomarker outcome and AFI status of the corresponding endoscopic area. Aneuploidy, 17p LOH, p53 IHC and cyclin A significantly associated with AFI positivity (p<0.05) (see online supplementary table S1). Since dysplasia could represent a confounding factor, we looked at this association after exclusion of dysplastic areas and found that aneuploidy and p53 IHC retained a significant association with AFI positivity (see online supplementary table S1). To confirm the clinical utility of this association, we compared the accuracy for an overall diagnosis of HGD/EC of the three-biomarker panel in AFI+ areas versus AFI− areas (figure 3). Notably, average AUCs in all six databases were significantly higher for the three-biomarker panel assessed on AFI+ areas compared with AFI− areas. We therefore concluded that AFI positivity is associated with an enrichment of biomarkers and is a suitable tool to guide biopsies for biomarkers.

**Figure 4:**
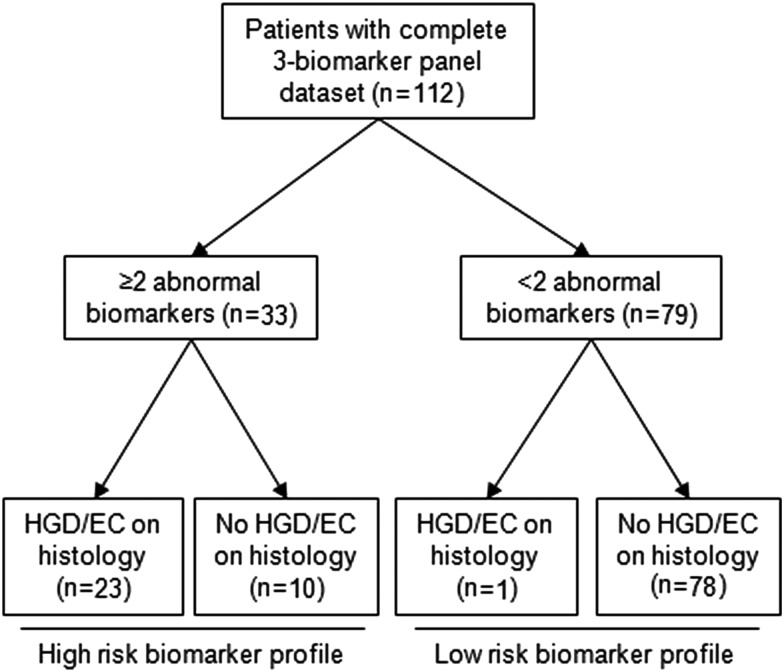
Flow chart of biomarker outcome in patient from the training cohort with full three-biomarker data set. Using a cut-off of two abnormal biomarkers to diagnose patients with prevalent high-grade dysplasia/early cancer (HGD/EC), the three-biomarker panel only missed one patient with HGD/EC. Ten patients with non-dysplastic Barrett's oesophagus (NDBO) or LGD were classified as high risk for HGD/EC. The sensitivity and specificity of three-biomarker panel for a diagnosis of HGD/EC were 95.8% (95% CI 76.9% to 99.8%) and 88.6% (95% CI 79.7% to 94.1%), respectively. LCD, low-grade dysplasia.

### Internal validation of the three-biomarker panel (per-patient analysis)

We sought to confirm that the biomarker panel selected in the per-biopsy analysis could correctly classify patients based on their highest grade of histological dysplasia. From a pool of 9 biomarkers, there are 84 different combinations of 3 biomarkers and 36 combinations of 2 biomarkers. We calculated the diagnostic accuracy of these 120 biomarker panels in the 2000 bootstrap samples generated from the original database. Strikingly, the best diagnostic accuracy was achieved by the same panel identified in the per-biopsy analysis (p53 IHC, cyclin A IHC and aneuploidy), with a cut-off of two positive biomarkers (see online supplementary table S2). We therefore took this panel forward to determine histological outcome in the patients from the training cohort that had the three-biomarker full data set (n=112). In keeping with the results from bootstrap resampling, a cut-off of two biomarkers positive had the best accuracy (see online supplementary table S3). Using this cut-off, only one patient with HGD was misclassified with a low-risk biomarker signature (figure 4 and table 3). The biomarker panel had a sensitivity and a specificity of 95.8% (95% CI 76.9% to 99.8%) and 88.6% (95% CI 79.7% to 94.1%), respectively, for a diagnosis of HGD/EC, and 74.4% (95% CI 57.6% to 86.4%) and 94.5% (95% CI 85.8% to 98.2%), respectively, for a diagnosis of any grade of dysplasia. By comparison, the Seattle protocol had similar sensitivities, namely, 95.8% (95% CI 76.9% to 99.8%) for a diagnosis of HGD/EC (p=1.0 when compared with three-biomarker panel) and 84.6% (95% CI 68.8% to 93.6%) for a diagnosis of any grade of dysplasia (p=0.26) (table 3). Relying on the histology of AFI+ areas, the sensitivity for HGD/EC was lower at 91.7%, although this difference was not statistically significant. Importantly, using this novel approach 2.8 biopsies per patient were taken on average compared with 12.8 for the standard biopsy protocol (p<0.001) (table 3).

**Table 3:** Comparison among Seattle protocol, AFI-targeted histology and three-biomarker panel on AFI+ areas

	Seattle protocol+histology	AFI+histology	AFI+biomarkers	p Value
No. of HGD/EC missed	1	2	1	N/A
Sensitivity for HGD/EC	95.8%	91.7%	95.8%	ns
Total no. of biopsies	1385	169	310	N/A
No of biopsies per patient	12.4	1.5	2.8	<0.001
No of biopsies for every HGD/EC case diagnosed	60.2	7.7	13.5	N/A

Seattle protocol includes all biopsies taken on HRE white light endoscopy (random + targeted on macroscopically visible abnormal areas). AFI+ histology includes biopsies taken on AFI+ areas, regardless of their appearance on HRE. AFI + biomarkers column includes only the biopsies from AFI+ areas processed for the three-biomarker panel.

AFI, autofluorescence imaging; EC, early cancer; HGD, high-grade dysplasia; HRE, high-resolution endoscopy.

### External validation of the three-biomarker panel

We then tested the biomarker panel in an independent group of 46 patients. The panel had a sensitivity and a specificity of 100% and 85% (95% CI 98.9% to 95.0%), respectively, for a diagnosis of HGD/EC, and 73.9% (95% CI 51.6% to 89.7%) and 100%, respectively, for a diagnosis of any grade of dysplasia (see online supplementary table S4). The Seattle protocol missed two cases of HGD/EC, which translated into a sensitivity of 83.3% for a diagnosis of HGD/EC (p=0.14 when compared with three-biomarker panel) and 91.3% for a diagnosis of any grade of dysplasia (p=0.12), but relied on a significantly higher number of biopsies (p<0.001).

## Discussion

In the present study, we devised a novel strategy in which advanced endoscopic imaging can be used in BO to direct biopsy sampling for biomarkers. Single biomarkers or panels of biomarkers have been shown to correlate with dysplasia^20^ ^22^ ^23^ ^35^ and in some cases to estimate the individual risk of progression.^22^ ^23^ ^35–37^ This prospective multicentre phase IV study evaluated nine biomarkers with the most robust published data, and, to our knowledge, is the first study that has validated such a large panel in a single patient cohort and in an independent laboratory. The rationale for using a biomarker panel stems from evidence that a combination of biomarkers performs better than single biomarkers on their own.^35^ Clinical applicability of biomarkers relies on the feasibility of the specific laboratory assays, low enough costs and a manageable number of assays if combined into a panel. The panel identified in this study satisfies these criteria. p53 and cyclin A rely on IHC, which is a routine technique in pathology laboratories. Of these, p53 is currently already employed by some pathologists to aid in the diagnosis of dysplasia^19^ ^20^ and has been recommended in the revised British guidelines.^38^ For the evaluation of aneuploidy, we used flow cytometry on frozen samples, as previously published.^35^ For future clinical applications, image cytometry (IC) is an alternative technique applicable to paraffin sections and has been recently shown to be comparable to flow cytometry in BO.^39^ Hence, in the future, having refined the number of biomarkers from 9 to 3, the panel can be applied from a single diagnostic biopsy stored in formalin.

In this study, we hypothesised that AFI positivity might correlate with a field of molecular abnormality related to dysplasia in BO. Indeed, we found that aneuploidy and p53 abnormalities significantly correlated with AFI positivity independently of dysplasia within that AFI+ area. DNA is known to be a weak fluorophore; therefore, the loss of autofluorescence may be explained by the increased DNA content observed in aneuploidy. Similarly, *p53* is a key gene in the control of DNA amplification and proliferation of aneuploid cells,^40^ whereas cyclin A can be overexpressed as result of cell cycle deregulation leading to aneuploidy.^41^ Inflammation has previously been shown to associate with AFI false positivity.^14^ Since inflammatory pathways can drive genetic and epigenetic changes during carcinogenesis in BO,^42^ it is possible that the AFI signal, inflammation and biomarkers are all closely related.

Assessment of dysplasia in the AFI-targeted areas relied only on diagnostic biopsies stored in formalin; therefore, there is a possibility of sampling error for focal dysplasia. To reduce this possibility, large AFI+ areas had two or more diagnostic biopsies taken. Since the molecular changes precede development of dysplasia and expand over large areas of mucosa,^24^ we believe that the field of molecular abnormality is less affected by sampling error.

Notably, the new diagnostic tool had the same sensitivity for dysplasia as the Seattle protocol with significantly fewer biopsies. Therefore, this novel risk stratification approach, which is based on more objectively measurable outcomes, has the potential to overcome several of the major limitations of BO surveillance endoscopy, including sampling error and the subjectivity of a dysplasia diagnosis. Four pathologists with a special interest in BO took part in this study, and still the agreement for a diagnosis of any grade of dysplasia was ‘moderate’. Hence, a binary outcome of a molecular test with a predetermined cut-off value can prove advantageous in this regard. In addition, the small number of biopsies may lead to shorter endoscopic procedures and a lower risk of complications. From an economic perspective, we estimated that the costs required to assess the three-biomarker panel (aneuploidy by IC + IHC for p53 and cyclin A) on biopsies targeted by AFI were similar to the costs of the Seattle protocol, and these costs would come down with high-throughput assay techniques (data not shown).

This is a cross-sectional study to diagnose prevalent dysplasia, and therefore we did not set out to analyse follow-up data. However, it is notable that among the 10 patients in the training cohort with a high-risk biomarker profile (≥2 abnormal biomarkers) and no evidence of HGD/EC at the time of the endoscopy (figure 4), 6 of them (60%) had pathological progression within 6 months (4 from low-grade dysplasia (LGD) to HGD/EC, 1 from ID to EC and 1 from ID to LGD). Assuming this could have been prevalent disease missed by sampling error, this confirms that biomarkers overcome this limitation of the Seattle protocol. However, the lack of follow-up data did not allow us to draw conclusions concerning the clinical implications of the novel method, especially in relation to the role of this approach for predicting future cancer progression as compared with the current standard of practice.

This study has several strengths. It is the first study designed prospectively to assess a predefined group of multiple biomarkers in a large cohort of patients with BO and by a single laboratory. To our knowledge, this is the largest imaging study in BO and it has a sample size comparable to other biomarker studies.^23^ ^35^ Finally, the endoscopic protocol was carefully monitored to guarantee proper training and strict adherence.

This study has some limitations. Only approximately 70% of patients could enter the per-patient analysis in the original database (112 out of 157). This is due to the fact that at the start of the study we lacked information about which of the nine biomarkers would outperform the others. Therefore, since not all the AFI+ areas were large enough to allow for three research biopsies, we evenly distributed different biomarker analyses among the available biopsies and then used a combination of MI and bootstrap resampling to deal with missing data.^34^ By contrast, all patients included in the validation cohort could be fully assessed with the three biomarkers, suggesting that a smaller panel is clinically feasible. Furthermore, the patient cohorts do not reflect the general BO population as enrichment for high-risk patients was necessary to guarantee an adequate number of pathological outcomes to correlate with biomarker positivity. Moreover, we cannot exclude that the improved diagnostic accuracy of biomarkers on AFI+ areas (figure 3) is influenced by the higher number of AFI+ areas sampled compared with AFI− regions. In order to maximise the molecular information related to AFI positivity, we aimed to sample at most four AFI+ and one AFI− area per patient. However, the ratio between AFI+ and AFI− areas was 1.6:1 in the training cohort and 2.3:1 in the validation cohort, suggesting that this only had a small impact on the final results. In addition, although AFI is not compatible with all the endoscopic technologies available across the world, we believe that this study has paved the way for a new diagnostic approach based on the integration of endoscopic and molecular data, and in the future other advanced imaging modalities could be used to target biopsies for biomarker assessment. The advantage of AFI is that, like chromoendoscopy, the assessment of a colour-coded image is relatively easy and our data on the reliability of AFI signal interpretation confirmed a good level of agreement among endoscopists. Finally, although the exclusion of patients with short segments makes this study mostly relevant to patients with long segment of BO, we believe that the latter group of patients is the one that particularly benefits from red-flag endoscopic techniques and biomarkers due to the higher rate of sampling error and number of biopsies compared with short segments of BO.

In conclusion, this study provides evidence that a three-biomarker panel on AFI-targeted biopsies has equivalent diagnostic accuracy for dysplasia compared with the current gold standard with a significant reduction in the number of biopsies required.

## Supplementary Material

Web supplement
