# Supplementary material for: The combination of autofluorescence endoscopy and molecular biomarkers is a novel diagnostic tool for dysplasia in Barrett's oesophagus
Source: Gut. 2014 Apr 10;64(1):49–56. doi: 10.1136/gutjnl-2013-305975 (PMC4283667; doi:10.1136/gutjnl-2013-305975)

SUPPLEMENTARY TEXT AND TABLES

Biomarker selection

A PubMed search was conducted with the following search terms: Barrett's (o)esophagus and biomarker and dysplasia. This retrieved 345 publications of which 48 were original articles on molecular biomarkers. In depth analysis was conducted on the 26 studies, of which 2 related to molecular biomarker panels. We selected 6 Phase III/IV biomarker studies which showed robust data on a total of 9 molecular biomarkers and their correlation with dysplasia in BO. These 9 biomarkers were included in the study

Flow cytometry

The snap frozen biopsies in DMSO were used for analysis of DNA content abnormalities and processed as described previously.(1) The isolated nuclei were analyzed by flow cytometry using a MoFlow (Beckman Coulter, Miami, FL, USA) or BD Influx™ (Becton, Dickenson biosciences, New Jersey, USA) by an expert cytometrist. The cell cycle histogram was analyzed using ModFit LT (Verity Software House, Topsham, ME, USA) by two investigators, who were blind to the histological outcome, and discordant results were reviewed jointly to reach agreement.

Quantitative methylation-specific PCR (Methylight)

DNA was extracted from snap frozen esophageal biopsies using the DNeasy® Blood & Tissue Kit (Qiagen, Hilden, Germany) according to the manufacturer's instructions. DNA was bisulfite modified using an EpiTect® Bisulfite Kit (Qiagen) and the degree of methylation was analyzed by Methylight using a LightCycler® 480 (Roche diagnostics Ltd, Rotkreuz, Switzerland) with previously published Taqman primers and probes.(2) Normalized methylation values were calculated as previously described.(2)

Immunohistochemistry (IHC)

Staining was performed with the BOND™ System (Leica Microsystems, Ltd, Milton Keynes, UK) with an H1 antigen retrieval program. Antibodies used were: p53 clone DO7 (Dakocytomation, 1:50) and cyclin A (Novocastra, 1:40). The scoring was performed by a single author without prior knowledge of the clinical diagnosis. Surface cyclin A was scored as previously described, using a cut-off for

positivity of 1% of positive surface cells.(3) P53 expression was scored positive when there were areas of strong staining or complete loss of staining, compared to the background levels, as previously described.(4)

Loss of heterozygosity (LOH)

LOH at 17p and 9p loci (*p53* and *p16* genes, respectively) was assessed on DNA extracted by phenol/chloroform. Microsatellite markers were used as previously published.(5) The microsatellite loci were amplified by multiplex PCR and the PCR products were combined with GeneScan™ LIZ® Size Standard and electrophoresed on an automated sequencing system (ABI 3130 xl Genetic Analyzer, Applied Biosystems, California, USA) according to the manufacturer's protocol. GeneMapper® Software (Applied Biosystems) was used to analyse the peak height ratios of the alleles of a duodenal control and oesophageal biopsy and highlight LOH candidates. LOH was assessed on the basis of the following allelic imbalance ratios:

$$\text{Allelic ratio of sample} = \frac{\text{Peak height of Allele 1}}{\text{Peak height of Allele 2}}$$

$$\text{Allelic Imbalance} = \frac{\text{Allele Ratio of Control Sample}}{\text{Allele Ratio of AFI Sample}}$$

Ratio of > 1.35 or < 0.067 was considered as suggestive of LOH as instructed by the software. The sample was considered positive for LOH if ≥2 loci were suggestive for LOH for a particular gene.

Statistical methods

In order to identify a biomarker panel that could predict histological outcome, we used a bootstrap resampling method(6) and took 100 bootstrap samples for each MI dataset and the original database. For each bootstrap sample, we tested individually the 512 different combinations of 9 biomarkers for their association with HGD/EC. Out of the 512 possible models, the best one was selected according to the Akaike information criterion (AIC) of a logistic regression model. In order to correct for potential patient effects when multiple AFI-targeted areas derived from the same patient, they were included as random effects in the model. Adding such effect assumes that, in addition to other explanatory variable,s there might be an extra factor common to all samples from the same patient. However,

since we were not interested in a specific fixed patient effect, but focused on population level inference, it was assumed to be a random effect and integrated over using the glmer function of the lme4 R package.(7) For each biomarker we calculated the bootstrap inclusion frequency, which was defined as the number of times the biomarker was selected in the 100 best models. The biomarkers with a median inclusion frequency of at least 90 over the imputed datasets and original database were selected for the biomarker panel.(8) The predicted probability of dysplasia for each endoscopic area obtained from a logistic regression with the selected biomarker panel as predictor was used to generate the receiver operating characteristic (ROC) curves to evaluate the diagnostic accuracy of the panel and the area under the ROC curve (AUC) was reported.

For the purpose of the per-patient analysis, in patients with at least one AFI+ area and one AFI- area two datasets, called AFI+ and AFI- dataset, were created. In the AFI+ dataset, each biomarker was assigned a positive outcome, if the biomarker was positive in at least one AFI+ area, and negative if negative in all AFI+ areas. An identical process was followed for the AFI- dataset. The histological diagnosis for a patient was the overall histology (see biopsy and histology section). A logistic regression model, with the selected biomarker panel as predictor, was fitted using patients from a bootstrap sample and was then validated in the patients that were not selected in the bootstrap sample. This process was repeated 2000 times separately for the AFI+ and the AFI- datasets, resulting in 2000 AUC values for each dataset, which were compared in a paired t-test pairing bootstrap samples from the AFI+ and AFI- datasets. This comparison was done in all the 5 imputed databases and the original database.

To validate the biomarker panel selected in the per-biopsy analysis in the per-patient analysis, we applied a simple counting approach to the original database. For each possible panel of two or three biomarkers, a patient was predicted as HGD or EC if the number of positive biomarkers in the panel reached a certain cut-off point. We calculated sensitivity and specificity of each panel for a diagnosis for HGD/EC based on different cut-off points. The best biomarker panel was defined as the one with the highest accuracy (average of sensitivity and specificity). This process was repeated in 2000 bootstrap samples from the original database and the mean and standard deviation of the accuracy was reported for each panel at different cut-off points.

Bibliography

1. Reid BJ, Haggitt RC, Rubin CE*, et al.* Barrett's esophagus. Correlation between flow cytometry and histology in detection of patients at risk for adenocarcinoma. *Gastroenterology* 1987;**93**:1-11.
2. Schulmann K, Sterian A, Berki A*, et al.* Inactivation of p16, RUNX3, and HPP1 occurs early in Barrett's-associated neoplastic progression and predicts progression risk. *Oncogene* 2005;**24**:4138-48.
3. Lao-Sirieix P, Lovat L, Fitzgerald RC. Cyclin A immunocytology as a risk stratification tool for Barrett's esophagus surveillance. *Clin Cancer Res* 2007;**13**:659-65.
4. Depledge DP, Evans KJ, Ivens AC*, et al.* Comparative expression profiling of Leishmania: modulation in gene expression between species and in different host genetic backgrounds. *PLoS Negl Trop Dis* 2009;**3**:e476.
5. Galipeau PC, Li X, Blount PL*, et al.* NSAIDs modulate CDKN2A, TP53, and DNA content risk for progression to esophageal adenocarcinoma. *PLoS Med* 2007;**4**:e67.
6. Sauerbrei W. The use of resampling methods to simplify regression models in medical statistics. *Appl Statist* 1999;**48**:313-29.
7. Pinheiro JCaB, D.M. Mixed-effects Models in S and S-PLUS. *New York: Springer* 2000.
8. Sauerbrei W. The use of resampling methods to simplify regression models in medical statistics. *Journal of the Royal Statistical Society: Series C (Applied Statistics)* 1998;**48**:313-29.

Supplementary Table 1. Association of molecular biomarkers with AFI status

Biomarker	Biomarker outcome	All areas			Non dysplastic areas		
		AFI-	AFI+	P	AFI-	AFI+	P
HPP1 methylation	Negative	30 (23.8%)	32 (18.3%)	0.25	29 (25.2%)	27 (22.9%)	0.76
	Positive	96 (76.2%)	143 (81.7%)		86 (74.8%)	91 (77.1%)	
RUNX3 methylation	Negative	50 (39.7%)	58 (33.1%)	0.27	49 (42.6%)	48 (40.7%)	0.79
	Positive	76 (60.3%)	117 (66.9%)		66 (57.4%)	70 (59.3%)	
P16 methylation	Negative	71 (56.3%)	84 (48.0%)	0.16	67 (58.3%)	65 (55.1%)	0.69
	Positive	55 (43.7%)	91 (52.0%)		48 (41.7%)	53 (44.9%)	
P53 IHC	Negative	96 (76.8%)	103 (52.0%)	<0.01	93 (83.0%)	96 (71.6%)	0.05
	Positive	29 (23.2%)	95 (48.0%)		19 (17.0%)	38 (28.4%)	
Cyclin A IHC	Negative	111 (84.1%)	135 (72.2%)	0.02	106 (89.1%)	117 (88.6%)	1.00
	Positive	21 (15.9%)	52 (27.8%)		13 (10.9%)	15 (11.4%)	
Tetraploidy	Negative	89 (70.6%)	114 (65.9%)	0.45	85 (74.6%)	86 (69.9%)	0.47
	Positive	37 (29.4%)	59 (34.1%)		29 (25.4%)	37 (30.1%)	
Aneuploidy	Negative	115 (91.3%)	128 (74.0%)	<0.01	109 (95.6%)	107 (87.0%)	0.02
	Positive	11 (8.7%)	45 (28%)		5 (4.4%)	16 (13.0%)	
17p LOH	Negative	60 (53.6%)	60 (38.0%)	0.01	57 (55.9%)	50 (48.1%)	0.27
	Positive	52 (46.4%)	98 (62.0%)		45 (44.1%)	54 (51.9%)	
9p LOH	Negative	19 (17.0%)	30 (19.5%)	0.63	17 (16.7%)	23 (22.3%)	0.38
	Positive	93 (83.0%)	124 (80.5%)		85 (83.3%)	80 (77.7%)	

Supplementary Table 2. Twenty best biomarker panels for the diagnosis of HGD/EC from a total pool of 84 different combinations of 3 biomarkers and 36 combinations of 2 biomarkers with different cut-off values. Cut-off refers to the number of abnormal biomarkers required to yield a positive outcome. For each cut off the means of the diagnostic accuracy form 2000 bootstrap samples and the standard deviation are represented. Highlighted in bold the best cut off for each panel.

			Mean			Standard deviation		
Biomarker 1	Biomarker 2	Biomarker 3	cut-off 1	cut-off 2	cut-off 3	cut-off 1	cut-off 2	cut-off 3
Cyclin A IHC	p53 IHC	aneuploidy	0.765272	**0.866055**	0.780642	0.025394	0.037145	0.048938
Cyclin A IHC	p53 IHC	tetraploidy	0.682272	**0.859118**	0.709647	0.024396	0.033878	0.051788
Cyclin A IHC	p53 IHC	P53 LOH	0.683017	**0.856624**	0.798002	0.024707	0.034406	0.047969
Cyclin A IHC	p53 IHC	p16 methyl	0.683315	**0.839828**	0.76563	0.024397	0.034727	0.048326
p53 IHC	tetraploidy	P53 LOH	0.635249	**0.836269**	0.65821	0.024351	0.036256	0.053326
Cyclin A IHC	p53 IHC	N/A	0.778035	**0.83559**	N/A	0.023653	0.040751	N/A
Cyclin A IHC	tetraploidy	aneuploidy	0.707605	**0.835231**	0.658476	0.030779	0.042051	0.047343
Cyclin A IHC	p53 IHC	RUNX3 methyl	0.632646	0.799535	**0.834621**	0.022021	0.035843	0.044334
Cyclin A IHC	aneuploidy	RUNX3 methyl	0.641786	**0.832034**	0.752845	0.023248	0.040472	0.050709
Cyclin A IHC	p53 IHC	p16 LOH	0.599525	**0.827687**	0.793979	0.020432	0.025124	0.047418
Cyclin A IHC	aneuploidy	P53 LOH	0.679156	**0.826015**	0.712769	0.038479	0.045307	0.050269
p53 IHC	RUNX3 methyl	N/A	0.632093	**0.811182**	N/A	0.028636	0.040024	N/A
Cyclin A IHC	RUNX3 methyl	N/A	0.646782	**0.808857**	N/A	0.028894	0.044359	N/A
p53 IHC	HPP1 methyl	P53 LOH	0.537662	0.706159	**0.806901**	0.013877	0.034944	0.046313
p53 IHC	p53 LOH	N/A	0.71064	**0.801271**	N/A	0.030795	0.045302	N/A
Cyclin A IHC	p53 IHC	HPP1 methyl	0.540834	0.815344	**0.805547**	0.013671	0.024707	0.046789
Cyclin A IHC	p53 IHC	P53 LOH	0.683017	0.856624	**0.798002**	0.024707	0.034406	0.047969
p53 IHC	RUNX3 methyl	P53 LOH	0.609351	0.778615	**0.795858**	0.021577	0.03136	0.046959
Cyclin A IHC	p53 IHC	P53 LOH	0.599525	0.827687	**0.793979**	0.020432	0.025124	0.047418
p53 IHC	aneuploidy	N/A	0.78885	**0.79205**	N/A	0.031008	0.049292	N/A

Supplementary Table 3. Detailed biomarker outcome in patients with a complete 3-biomaker panel dataset in the training cohort

Number of positive biomarkers	Number of patients with NDBO/LGD (%)	Number of patients with HGD/EC (%)	Total of patients
0	50 (100.0%)	0 (0)	50
1	28 (96.6%)	1 (3.4%)	29
2	8 (53.3%)	7 (46.7%)	15
3	2 (11.1%)	16 (88.9%)	18
Total of patients	88	24	112

Supplementary Table 4. Detailed biomarker outcome in patients with a complete 3-biomaker panel dataset in the validation cohort.

Outcome of the 3-biomarker panel	Number of patients with NDBO/LGD (%)	Number of patients with HGD/EC (%)	Total of patients
Negative (<2 positive biomarkers)	29 (85.0%)	0 (0%)	29
Positive (≥2 positive biomarkers)	5 (15.0%)	12 (100.0%)	17
Total of patients	34	12	46

Supplementary Figure 1. Endoscopic imaging and biopsy protocols. A. Flowchart of endoscopic imaging and biopsy protocol. B. Examples of endoscopic view on high resolution endoscopy (HRE) and autofluorescence imaging (AFI) in patients with no AFI+ areas (left), small AFI+ areas (middle) and large/complex AFI+ areas (right)

Supplementary Figure 1

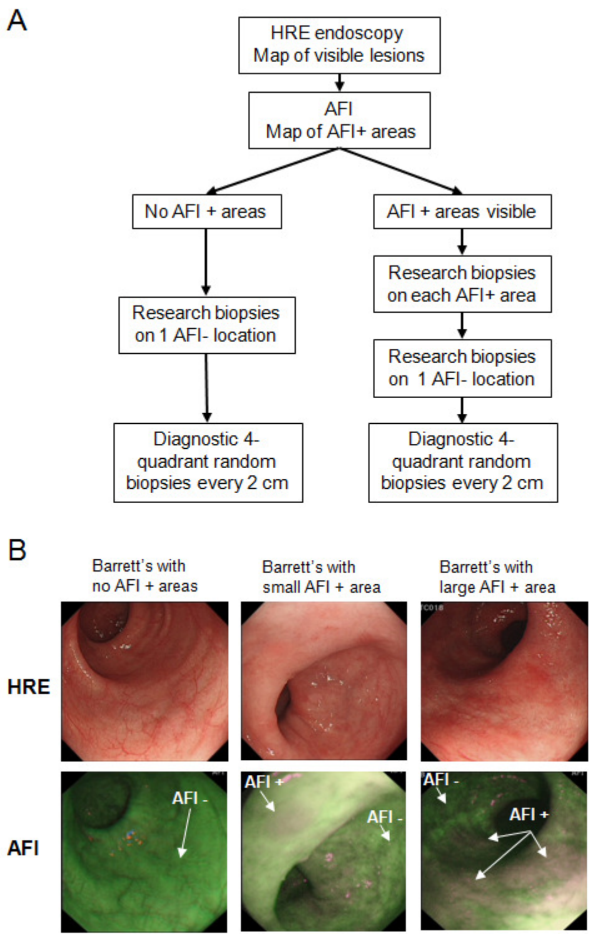

Supplement: Web supplement [file gutjnl-2013-305975-s1.pdf]
